# The effect of Internet use on adolescent nutritional outcomes: evidence from China

**DOI:** 10.1186/s41043-025-00856-9

**Published:** 2025-04-26

**Authors:** Weigang Liu, Yanjun Ren, Jian Liu, Jens-Peter Loy

**Affiliations:** 1 Department of Agricultural Economics, Kiel University, Kiel, Germany; 2 College of Economics and Management, Northwest A&F University, Shaanxi, China; 3 Department of Agricultural Markets, Leibniz Institute of Agricultural Development in Transition Economies (IAMO), Halle, Germany; 4 Sino-German Center for Agricultural and Food Economics, Northwest A&F University, Shaanxi, China

**Keywords:** Internet use, Nutritional outcomes, BMI-for-age z-score, Overweight

## Abstract

**Background:**

The increasing prevalence of adolescent overweight and obesity poses significant public health challenges, particularly in China. With the rapid adoption of the Internet, adolescents’ lifestyles, including dietary habits and physical activity levels, have undergone substantial changes. However, the causal relationship between Internet use and adolescent nutritional outcomes, especially in transitional economies, remains underexplored.

**Methods:**

This study employs longitudinal data from the China Health and Nutrition Survey (CHNS) and China Family Panel Studies (CFPS) to investigate the impact of Internet use on adolescents’ BMI-for-age z-scores and overweight status. An endogenous switching regression (ESR) model addresses potential self-selection bias. Heterogeneity analyses examine urban–rural and gender differences, while mechanism analyses identify dietary pathways influencing outcomes.

**Results:**

Internet use increases BMI-for-age z-scores and the likelihood of being overweight among adolescents, with more intensive Internet use further amplifying these effects. These effects hold across urban and rural areas as well as for both boys and girls, though the magnitude may vary. Mechanism analyses suggest that Internet use enhances protein intake while also leading to higher consumption of fast food and soft drinks.

**Conclusion:**

Internet use increases BMI-for-age z-scores and overweight risks among adolescents, reinforcing the need for targeted interventions to mitigate its negative health impacts. Policies promoting healthier online behaviors and better access to nutritional education are essential to ensuring that adolescents develop healthy lifestyle habits in the digital age. Addressing these challenges can help policymakers develop equitable health strategies for adolescents in transitional economies.

## Introduction

The prevalence of overweight among adolescents in both developed and developing countries is a growing concern, presenting significant challenges to public health. This issue is particularly evident in the rising costs associated with individual healthcare, health insurance, and public financial expenditures. Studies indicate that in 1975, less than 1% of adolescents aged 5–19 were obese. However, by 2016, over 124 million adolescents (6% of all girls and 8% of all boys) were obese, and more than 340 million adolescents aged 5–19 were overweight or obese [1]. The rapid increase in overweight adolescents significantly contributes to the financial burden on healthcare systems. Research shows that globally, childhood overweight and obesity result in an increased annual total medical cost of $237.55 per capita, with specific per capita increases of $56.52 for non-hospital healthcare, $14.27 for outpatient visits, $46.38 for medication, and $1975.06 for hospitalization [2]. Healthcare spending is 14% higher for those with diabetes and obesity than for those with diabetes without obesity [3]. In the US, each unit increase in adult body mass index (BMI) results in an additional $6 billion in annual public healthcare spending, and the obesity epidemic in 2009 led to an unnecessary expenditure of $148.2 billion [4]. In China, the prevalence of overweight and obesity among school-age children rose from 15.5% in 2010 to 24.2% in 2019, and the trend continued with an increase to 29.4% in 2022 [5].

Adolescent nutritional outcomes are influenced by several factors. Sedentary lifestyles and insufficient physical activity reduce energy expenditure, significantly contributing to overweight among adolescents [6, 7]. Dietary habits, particularly the frequent consumption of high-energy fast food and intake of high-sugar beverages, also play crucial roles in influencing adolescent BMI [8, 9]. Additionally, family socio-economic status and living environment are important factors affecting adolescents’ BMI [10, 11]. The impact of Internet use on adolescent nutritional outcomes, however, is mixed. Many studies indicate that excessive or problematic Internet usage is associated with a higher risk of overweight and obesity in adolescents [12, 13]. Furthermore, it contributes to mental health disorders and chronic conditions such as depression, dry eyes, vision impairment, and neck pain [14, 15]. Conversely, some studies suggest that Internet use does not significantly affect overweight in adolescents, particularly in girls [16, 17].

Internet use has experienced significant changes in transitional economics, yet the reasons why Internet use might impact adolescents’ nutritional outcomes remain unclear. Over the past 20 years, the Internet has evolved from a novel technology to an essential part of daily life, revolutionizing communication, commerce, and entertainment. The early 2000s saw a rapid expansion in Internet access, with broadband connections becoming widespread. Social media platforms, emerging in the mid-2000s, significantly changed how people interact and share information globally. In the 2010s, mobile Internet and smartphones further accelerated Internet usage, making it more accessible and integrated into everyday activities. For transitioning economies, the Internet has been a crucial tool for economic development, facilitating digital inclusion, innovation, and access to global markets, thereby fostering economic growth and social progress. The Internet has profoundly transformed the daily lives of adolescents, offering access to vast information, social connectivity, and diverse entertainment. Some cross-sectional studies show a significant positive association between Internet use and being overweight [13, 18], but several studies note that this phenomenon does not exist [19, 20]. However, all these studies focus on research in developed countries, with less attention given to developing countries, especially those in transition.

Turning our attention to China, research findings the impact of Internet usage on health remain mixed. Some studies find that Internet access reduces the likelihood of being overweight in adults and improves self-reported health, mental health, and daily living for older adults [21–24]. However, for adolescents, very few studies indicate that Internet access does not significantly impact academic performance, and no studies examine its impact on their nutritional health [25]. Most research on adolescent Internet use and nutrition-related outcomes lacks causal identification [26, 27], but some evidence suggests that excessive Internet use may be linked to unhealthy behaviors. In China, with its vast population, the rapid development of the Internet over the past 20 years has led to the rise of online learning, Internet games, online videos, and online shopping. The recent spread of mobile Internet significantly expands the ways adolescents use the Internet. Consequently, the Internet’s development in China has transformed how adolescents learn and entertain themselves, making it essential to further analyze the impact of their Internet use.

The Internet influences nutritional outcomes in adolescents through multiple channels. Some studies suggest that the Internet serves as a rich source of information, contributing to an increase in nutritional knowledge among young adolescents [28]. Remarkably, regardless of the adolescents’ weight status, it does not alter the frequency with which they access nutritional information online [29]. However, much of the current research focuses on the impact of Internet use on sedentary behavior and increased screen time, which in turn decreases sleep time and increases the risk of overweight and obesity [18, 30, 31]. Additionally, some studies show that adolescents who overuse the Internet may consume more beverages, eat less fruit, and possibly develop anorexia nervosa [32, 33].

Previous research has predominantly emphasized the correlation between Internet use and adolescent health. The analysis of mechanisms has been confined to the impact of Internet use on increasing sedentary time among adolescents, which subsequently leads to reduced energy expenditure and an increase in BMI. The focus has been on highlighting the adverse effects resulting from excessive Internet use and the associated risks of becoming engrossed in online activities. However, these studies lack thorough causal analysis and have not addressed inherent endogeneity issues, such as sample selection bias [34]. Previous research often overlooks the self-selection aspect of Internet use, which may be related to factors such as family income levels [35]. Moreover, few studies have focused on the impact of Internet access on adolescent health beyond investigating the adverse effects of excessive Internet use. Meanwhile, previous studies have typically used BMI as an indicator to measure adolescent nutrition outcomes [36]. However, this indicator is not well-suited to the physical condition of adolescents. The World Health Organization (WHO) suggests that BMI-for-age z-score is a more suitable indicator than BMI for assessing the growth and physical condition of adolescents aged 5–19 years because it accounts for age and gender, allowing for a more accurate comparison with standardized growth curves. This metric is tailored to the distinct growth patterns of adolescents, facilitating improved identification of nutritional concerns such as undernutrition or obesity.

This study provides insight into the causal effects of adolescent Internet use on BMI-for-age z-score. The main results demonstrate that Internet use significantly increases adolescent BMI and raises the risk of adolescents being overweight. This effect intensifies with increased time spent using the Internet. By examining adolescents’ nutritional intake and dietary patterns, the study reveals a notable rise in protein intake, alongside a significant increase in fast food and soft drink consumption.

This study makes several key contributions to the understanding of how Internet use affects adolescent nutritional outcomes. First, our study provides a more precise definition of both the independent and dependent variables in the context of adolescent Internet use and nutritional health. Unlike studies that approximate Internet exposure using policy-driven broadband expansion [24], we directly measure individual Internet use, encompassing both mobile and computer-based access. Additionally, we distinguish between general Internet users and excessive users, providing a more refined analysis of usage intensity. For the dependent variable, we employ BMI-for-age z-score and overweight status, both of which are specifically designed to assess adolescent growth and nutritional status. Compared to previous studies that rely on standard BMI cutoffs developed for adults, our approach ensures a more accurate representation of adolescent body composition and weight status.

Second, this study makes a methodological contribution by addressing self-selection bias in estimating the effects of adolescent Internet use on nutritional outcomes. We employ the Endogenous Switching Regression model, which not only accounts for selection effects but also controls for unobservable factors that may influence both Internet use and nutritional health. Compared to prior research that primarily establishes correlational relationships between Internet use and adolescent nutrition, our study provides a more rigorous analytical framework. To further validate our findings, we implement multiple robustness checks, including the Mundlak correction to account for unobserved individual effect and an alternative instrumental variable (IV) estimation strategy to address endogeneity concerns. These robustness tests confirm the stability and consistency of our results.

Third, this study integrates two large-scale longitudinal datasets, China Health and Nutrition Survey (CHNS) and China Family Panel Studies (CFPS), to construct a comprehensive empirical analysis. By leveraging data from both sources, we incorporate a broader and more representative sample, enhancing the generalizability and credibility of our findings. Moreover, the study extends the sample period to 2022, allowing us to capture the most recent trends in adolescent Internet use and its implications for nutritional health.

Finally, our study provides mechanism-based insights by examining how Internet use affects adolescent dietary behaviors. We analyze the impact of Internet use on both food consumption patterns and nutrient intake, offering a detailed understanding of how digital engagement translates into changes in dietary habits. This contributes to a more nuanced discussion on the role of the Internet in shaping adolescent nutrition beyond its overall impact on BMI-for-age z-score outcomes.

The subsequent sections of the study are organized as follows. The second section focuses on the methodology and data utilized, detailing the sample profile and the variable settings employed. The third section presents the empirical results, including an analysis of the main findings, robustness tests, heterogeneity analysis, and mechanism analysis. The final sections include a discussion, conclusions, and policy implications.

## Method

### Model

#### Endogenous switching regression model

Addressing self-selection bias in adolescents’ Internet use is crucial, as differences between Internet users and non-users may stem from both observable factors such as family economic status, geographical location, and household structure, and unobservable factors such as cultural influences, family Internet habits, technological proficiency, and individual preferences. If these differences are not properly accounted for, estimates of the relationship between Internet use and adolescent nutritional health may be biased, leading to misleading conclusions.

Several alternative methods, such as Propensity Score Matching (PSM) and Fixed Effects (FE) models, are commonly used to mitigate endogeneity concerns. However, these approaches have notable limitations in the context of this study. PSM relies on observable characteristics to construct comparable treatment and control groups, but it does not fully address unobserved heterogeneity that may simultaneously influence Internet use and nutritional outcomes. FE models control for time-invariant unobserved heterogeneity by eliminating individual-specific effects, but they do not account for time-varying unobservable factors that may simultaneously affect both Internet use and nutritional outcomes. Additionally, FE models assume that both observed and unobserved factors have homogeneous effects across individuals, which may not hold in this context, as the impact of Internet use on nutrition-related health may vary across different adolescent subgroups.

Given these limitations, we employ the Endogenous Switching Regression (ESR) model, which is particularly well-suited for this study for several reasons. First, ESR explicitly models the selection process, estimating separate outcome equations for Internet users and non-users to account for self-selection bias. Second, ESR provides a more flexible framework for capturing individual heterogeneity by allowing the effects of Internet use to vary across groups. Unlike PSM, which relies only on observable characteristics, or FE models, which assume homogeneous effects and fail to account for time-varying unobserved factors, ESR corrects for selection bias by incorporating correlated error structures between the selection and outcome equations. This ensures a more robust estimation of the impact of Internet use on adolescent nutritional outcomes. Third, by incorporating an IV approach, ESR strengthens causal inference by mitigating potential endogeneity concerns that arise from reverse causality. Finally, ESR is particularly useful for analyzing unbalanced panel data, as it allows us to estimate the effects of Internet use while accommodating variations in individual participation across survey waves.

We implement the methodology as follows. The ESR model involves two key steps First, we address the selection process inherent in adolescents’ decision to use the Internet. According to utility maximization theory, the decision to use the Internet is based on comparing expected utilities. Specifically, adolescents choose to use the Internet $\left( {F_{i} = 1} \right)$ if the expected utility of using the Internet $\left( {F_{1i}^{*} } \right)$ exceeds the expected utility of not using it $\left( {F_{0i}^{*} } \right)$. Since the expected utilities are unobservable, we use a latent variable model to represent this decision-making process.

1 $$F_{{{\text{it}}}}^{*} = \varphi Q_{it} + \rho I_{it} + \delta_{t} + \theta_{r} + \mu_{i} ,\;F_{i} = \left\{ {\begin{array}{*{20}l} 1 \hfill & {{\text{if}}\;F_{i}^{*} > 0} \hfill \\ 0 \hfill & {{\text{otherwise}}} \hfill \\ \end{array} } \right.$$

$F_{{{\text{it}}}}^{*}$ is the latent variable representing the underlying probability of an adolescent using the Internet. It is determined by the binary variable $F_{{{\text{it}}}}^{*}$ which equals 1 if the individual is an Internet user and 0 if the individual is a non-Internet user. $Q_{i}$ represents the relevant factors influencing the decision to use the Internet, $I_{it}$ is a vector of IVs serving as proxies for the independent variable of Internet use. $\delta_{t}$ represents year fixed effects, accounting for time-specific shocks affecting all individuals. $\theta_{r}$ represents regional fixed effects, controlling for location-based heterogeneity, and $\mu_{i}$ is the error term.

The relationship between Internet use and BMI-for-age z-score is potentially subject to reverse causality, making IV estimation necessary. Internet use may influence BMI-for-age z-score, as longer screen time could lead to more sedentary behavior, potentially contributing to weight gain. Conversely, BMI-for-age z-score could also influence Internet use, as adolescents with higher BMI-for-age z-score may prefer online entertainment over outdoor activities due to physical activity constraints. Given this bidirectional relationship, a valid IV is required to disentangle the causal effects. The IV is applied only in the first stage, and it is not included as a control variable in the second stage.

The second step in the ESR model involves estimating the determinants of BMI-for-age z-score and overweight in adolescents. To accommodate the different types of outcome variables, we use distinct estimation methods. For the continuous outcome variable (BMI-for-age z-score), we apply Ordinary Least Squares for estimation. For the binary outcome variable (overweight), we employ a Probit model for estimation. This is illustrated in Eq. 2 and Eq. 3. In Eq. 2, $Y_{{{\text{ait}}}}$ represents the outcome variables for adolescents using the Internet, specifically BMI-for-age z-score and overweight. $X_{{{\text{ait}}}}$ denotes the relevant factors influencing these outcome variables, and $\varepsilon_{{{\text{ai}}}}$ is the error term. In Eq. 3, $Y_{{{\text{bit}}}}$ represents the outcome variables for adolescents who do not use the Internet, namely BMI-for-age z-score and overweight. $X_{{{\text{bit}}}}$ represents the relevant factors affecting these outcomes, and $\varepsilon_{bi}$ is the error term in the equation.

Outcome equation for adolescents using the Internet:

2 $$Y_{{{\text{ait}}}} = \gamma_{a} X_{{{\text{ait}}}} + \delta_{t} + \theta_{r} + \varepsilon_{{{\text{ai}}}} \;{\text{if}}\;F_{i} = 1$$

Outcome equation for adolescents who do not use the Internet:

3 $$Y_{{{\text{bit}}}} = \gamma_{b} X_{{{\text{bit}}}} + \delta_{t} + \theta_{r } + \varepsilon_{{{\text{bi}}}} \;{\text{if}}\;F_{i} = 0$$

While $X_{{{\text{it}}}}$ captures observable factors influencing BMI and overweight status, unobservable characteristics (e.g., health consciousness, risk preferences) may still correlate with both the selection equation and outcome equations, leading to selection bias. The ESR model corrects for this issue by treating selection bias as a missing variable problem and incorporating the inverse Mills ratio $\left( {\lambda_{i} } \right)$. The inverse Mills ratio $\left( {\lambda_{i} } \right)$ controls for selection bias from unobservable factors, with $\sigma_{\mu a}$ and $\sigma_{\mu b}$ as its corresponding coefficients in the outcome equations for Internet users and non-users, respectively. The ESR model also enables a formal interpretation of selection bias through the correlation coefficients:

$$rho_{1} = \frac{{\sigma_{\mu a} }}{{\sigma_{\mu } }},\;rho_{0} = \frac{{\sigma_{\mu b} }}{{\sigma_{\mu } }}$$

If $rho_{1}$ or $rho_{0}$ is statistically significant, it confirms the presence of selection bias driven by unobservable factors.

### Estimating treatment effects

In the framework proposed by Rubin [37] and the specific analysis process outlined by Lokshin and Sajaia [38], we utilize counterfactual analysis to derive the treatment effects. The expected values of the observable outcome variables are as follows.

Expectations of outcome variables for adolescents using the Internet (observed):

4 $$E\left[ {Y_{{{\text{ait}}}} D_{it} = 1} \right] = \beta_{a} X_{{{\text{ait}}}} + \sigma_{\mu a} \lambda_{ia} + \delta_{t} + \theta_{r}$$

Expectations of outcome variables in the absence of Internet use for adolescents who use the Internet (counterfactual):

5 $$E\left[ {Y_{{{\text{bit}}}} D_{i} = 1} \right] = \beta_{b} X_{{{\text{ait}}}} + \sigma_{\mu b} \lambda_{ia} + \delta_{t} + \theta_{r}$$

Using the four equations above, the average treatment effect on the treated (ATT) is shown below.

6 $$ATT_{i} = E\left[ {Y_{ai} tD_{i} = 1} \right] - E\left[ {Y_{{{\text{bit}}}} D_{i} = 1} \right] = X_{{{\text{ait}}}} \left( {\beta_{a} - \beta_{b} } \right) + \lambda_{ia} \left( {\sigma_{\mu a} - \sigma_{\mu b} } \right)$$

## Data

### Sample

In this study, we utilize two databases for empirical research. The first database is the CHNS, and the second is the CFPS. The CHNS is a large-scale microdata project funded and supported by the National Institutes of Health (NIH), the Carolina Population Center (CPC), the Chinese Centers for Disease Control and Prevention, among others. The survey covers 12 provinces and 3 municipalities, which vary widely in geography, economic development, and public resource indicators. A multi-stage random clustering process was employed to draw the survey sample for each province. Counties within each region were stratified by income levels (low, medium, and high), and the questionnaire comprised household, individual, and community surveys.

CFPS is a nationally representative biennial longitudinal survey of Chinese communities, households, and individuals initiated by the Institute of Social Science Survey (ISSS) at Peking University in 2010. It focuses on changes in China’s socio-economic, educational, familial, demographic, and health aspects. And CFPS focuses on changes in China’s socio-economic, educational, familial, demographic, and health aspects. To understand these changes comprehensively, both macro-level analyses of overall trends and micro-level studies of specific provinces and cities are necessary. The sampling design divides the 25 provinces and cities into two categories: large sample provinces, which include Liaoning, Shanghai, Henan, Guangdong, and Gansu, and small sample provinces, which comprise the remaining 20. This multi-stage probability sample employs implicit stratification, using official administrative divisions for the first two stages to capture the hierarchical and comprehensive nature of China’s administrative structure. Geographic representation and socio-economic status are key considerations in the sampling design, ensuring the sample accurately represents China’s diverse regions. This method involves selecting households using a random start and systematic sampling to ensure each sampled village or community achieves the target of 25 households.

Both CHNS and CFPS contain information on whether an adolescent uses the Internet, but only CFPS provides detailed data on leisure-time Internet usage, while CHNS includes dietary intake information, which is absent in CFPS. Given these dataset characteristics, we append the two datasets to maximize data coverage. To ensure consistency with WHO standards, our study focuses exclusively on adolescents aged 10 to 19. Consequently, we restrict the CHNS data to the 2006, 2009, and 2011 waves, as only these waves contain dietary intake information. The CFPS data include the 2010, 2014, 2016, 2018, 2020, and 2022 waves. After excluding provinces where the number of observations remained consistently below 10 per wave and removing missing values, the final dataset consists of 19, 317 observations, with 1, 834 from CHNS and 17, 483 from CFPS. Detailed information on the provincial and temporal composition of the dataset can be found in Appendix Table 9. For our analysis of whether Internet use affects adolescent BMI-for-age z-score, we utilize all available data from both CHNS and CFPS. However, when examining the effect of Internet usage time on BMI-for-age z-score, we rely solely on CFPS data, as CHNS does not contain this information. Similarly, when investigating the mechanisms underlying the relationship between Internet use and nutritional outcomes, we use only CHNS data, as it is the only dataset that provides dietary intake details.

### Variable

**Fig. 1:**
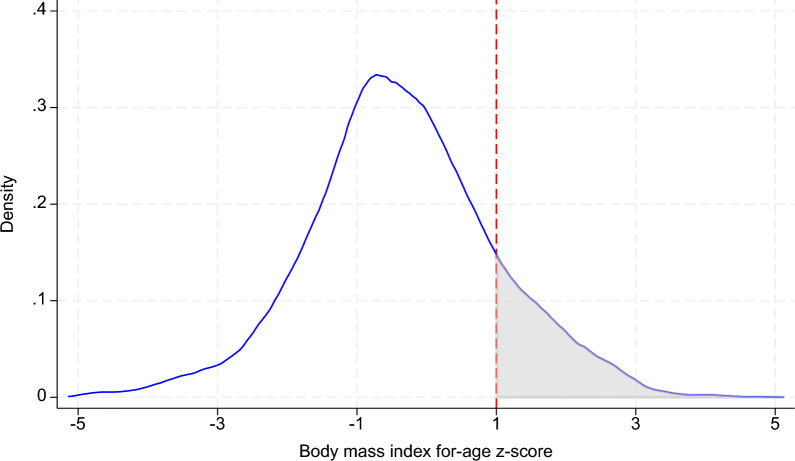
Distribution of BMI-for-age z-score. *Note*: BMI-for-age z-score equals 1 or above are defined as overweight. *Source*: CHNS 2006, 2009, 2011 and CFPS 2010, 2014, 2016, 2018, 2020, 2022

The definitions and descriptive statistics for the variables are presented in Table 1. The treatment variable, defined in the questionnaire as"Can you access the Internet,"indicates whether adolescents use the Internet. Specifically, the questionnaire also includes questions about whether they access the Internet via a computer or a mobile device. To measure adolescents’ health status, we analyze two dependent variables: BMI-for-age z-score and overweight status. The BMI-for-age z-score is calculated based on the WHO standards, following the methodology described by de Onis [39]. The range of the BMI-for-age z-score is between − 5 and 5. Drawing from existing definitions, this study categorizes BMI-for-age z-scores as follows: 1 to 1.99 as overweight, 2 to 2.99 as obese, and 3 or higher as severely obese [39, 40]. However, recognizing that overweight, obesity, and severe obesity all represent unhealthy nutritional outcomes, this research classifies any BMI-for-age z-score above 1 as indicative of overweight. As shown in Fig. 1, the distribution of BMI-for-age z-scores in the sample indicates a very high proportion of overweight adolescents, with more than 14% classified as overweight. Given China’s large population, the prevalence of overweight adolescents is particularly concerning.

**Table 1:** Descriptive statistics of the main variables *Sources:* The estimations are based on the CNHS 2006, 2009, 2011 and CFPS 2010, 2014, 2016, 2018, 2020, 2022

Variables	Definitions	All	Internet users	Non-users	Difference
		(1)	(2)	(3)	(4)
Internet	1 if use Internet, 0 otherwise	0.610	1.000	0.000	1.000
		(0.488)	(0.000)	(0.000)	(0.000)
Internet time	How many leisure hours per day are spent on the Internet	1.326	2.147	0.000	2.147***
		(2.269)	(2.563)	(0.000)	(0.031)
BMIZ	Body mass index for-age z-score	− 0.357	− 0.328	− 0.403	0.075***
		(1.339)	(1.255)	(1.460)	(0.020)
Overweight	If BMIZ > = 1, 0 otherwise	0.149	0.144	0.156	− 0.012**
		(0.356)	(0.351)	(0.363)	(0.005)
Age	Years	14.137	14.839	13.039	1.800***
		(2.826)	(2.755)	(2.575)	(0.040)
Gender	1 if boys, 0 means girls	0.525	0.543	0.497	0.046***
		(0.499)	(0.498)	(0.500)	(0.007)
Education	Years of education	7.491	8.344	6.154	2.191***
		(2.845)	(2.720)	(2.500)	(0.039)
Exercise	Number of exercises per week	3.280	3.550	2.856	0.695***
		(3.241)	(3.286)	(3.123)	(0.048)
Household size	Household size	4.798	4.630	5.060	− 0.430***
		(1.785)	(1.727)	(1.843)	(0.026)
Income	Natural logarithm of the per capita household income(yuan)	9.072	9.350	8.635	0.715***
		(1.135)	(1.062)	(1.107)	(0.016)
Urban	I if living in the urban area, 0 otherwise	0.330	0.410	0.204	0.206***
		(0.470)	(0.492)	(0.403)	(0.007)
County penetration	Internet penetration rate in local counties	0.439	0.513	0.322	0.192***
		(0.270)	(0.254)	(0.253)	(0.004)
Carbohydrate	Natural logarithm of 3-day average: Carbohydrate(g)	5.455	5.433	5.482	− 0.049***
		(0.382)	(0.387)	(0.375)	(0.018)
Fat	Natural logarithm of 3-day average: Fat(g)	4.041	4.174	3.886	0.288***
		(0.558)	(0.496)	(0.587)	(0.025)
Protein	Natural logarithm of 3-day average: Protein(g)	4.034	4.118	3.936	0.182***
		(0.363)	(0.347)	(0.357)	(0.016)
Fast food	How many times have you eaten fast food in the last three months?	1.232	1.790	0.580	1.210***
		(3.076)	(3.583)	(2.178)	(0.141)
Soft drink	Average liters drink per week	2.910	2.841	2.991	− 0.149*
		(1.663)	(1.512)	(1.822)	(0.078)
Observations		19, 317	11, 791	7, 526	19, 317

∗ ∗  ∗, ∗  ∗ and ∗ denote significance at the 1% level, 5% level and 10% level, respectively

Standard errors are presented in parentheses

The channel variables in our study are selected based on three aspects. First, nutrient intake: the CHNS surveys the food consumption of the population for three consecutive days and calculates nutrient intake. We focus on the intake of three nutrients significantly affecting adolescents’ BMI-for-age z-score: carbohydrates, proteins, and fats. According to Table 1, adolescents who use the Internet have lower carbohydrate intake, higher protein intake, and higher fat intake compared to those who do not use the Internet, with significant differences observed for all three variables. The second channel variable is the frequency of fast-food consumption, defined as the number of times adolescents consumed Western fast-food in the past three months. The third channel variable is soft drink intake, defined as the average weekly consumption of soft drinks in liters. Adolescents who use the Internet have significantly higher frequencies of Western fast-food consumption and soft drink intake compared to those who do not use the Internet.

When selecting IVs, two main factors are considered: correlation and exogeneity. Previous studies have used Internet penetration as an IV to assess rural development in Internet access and technology adoption [41, 42]. In this study, county-level Internet penetration is employed as an IV, satisfying the relevance criterion. To ensure accuracy, we calculate county-level Internet penetration based on actual Internet participation rates in each county during the respective survey wave, which effectively captures regional variations in Internet infrastructure at that time. Regarding instrument relevance, as reported in Table 10 in the Appendix, county-level Internet penetration is strongly predictive of Internet use, with a coefficient of 0.368. Additionally, the Kleibergen-Paap rk Wald F-statistic is 369.451, which rejects the null hypothesis of a weak instrument. Taken together, these results confirm that the IV is relevant. For instrument exogeneity, as shown in Table 11, we conducted reduced-form regressions to assess whether the IV directly influences BMI-for-age z-score. Results indicate that while county-level Internet penetration is significantly correlated with BMI in specifications without time fixed effects, the association becomes insignificant once time fixed effects are included. This confirms that county-level Internet penetration affects BMI only through Internet use, satisfying the exogeneity assumption.

Based on previous studies, the selected control variables include age, gender, educational level, and frequency of after-school sports participation at the individual level. Household-level factors include household size, household income per capita, and whether the residential address is urban or rural [43, 44]. According to the descriptive statistics provided in Table 1, Internet-using adolescents are older, more often boys, more educated, exercise more frequently, have smaller families, have higher family incomes, and are more likely to live in urban areas compared to non-Internet-using adolescents. Notably, adolescents who use the Internet exercise more than those who do not.

### Empirical Results

This section begins by presenting the estimation of the impact of Internet use on nutritional outcomes. The subsequent part conducts a heterogeneity analysis, followed by an examination of the mechanisms underlying the relationship between Internet use and nutritional health. The final part includes a robustness check.

### Estimation of Internet use on nutritional outcomes

In this comprehensive analysis employing the ESR model, the first-stage results illuminate the nuanced relationship between Internet use and nutritional outcomes, specifically BMI-for-age z-score and overweight, across distinct groups of individuals—Internet users and non-users. $rho_{1}$ and $rho_{0}$ are statistically significant, it confirms the presence of selection bias driven by unobservable factors. As shown in Table 2, the Wald/LR test of independent equations is used to assess whether the selection equation and the outcome equations are correlated, testing whether $rho_{1}$ and $rho_{0}$ are significantly different from zero. Prob > Chi2 is lower than 0.1, the null hypothesis of independence is rejected, indicating that the error terms of the selection and outcome equations are correlated, suggesting the presence of selection bias and supporting the use of the ESR model.

**Table 2:** Estimation of Internet use on nutritional outcomes *Sources*: The estimations are based on the CNHS 2006, 2009, 2011 and CFPS 2010, 2014, 2016, 2018, 2020, 2022

	BMIZ			Overweight		
	Selection	Internet users	Non-users	Selection	Internet users	Non-users
	(1)	(2)	(3)	(4)	(5)	(6)
Age	0.109***	− 0.072***	− 0.111***	0.107***	− 0.091***	− 0.162***
	(0.008)	(0.009)	(0.010)	(0.007)	(0.015)	(0.014)
Gender	0.139***	0.186***	0.209***	0.161***	0.480***	0.455***
	(0.022)	(0.022)	(0.035)	(0.021)	(0.031)	(0.045)
Education	0.131***	0.022***	− 0.072***	0.128***	0.029**	− 0.050***
	(0.008)	(0.008)	(0.010)	(0.008)	(0.014)	(0.015)
Exercise	0.014***	0.003	− 0.028***	0.013***	0.001	− 0.024***
	(0.004)	(0.004)	(0.006)	(0.004)	(0.005)	(0.007)
Household size	− 0.020***	− 0.019***	0.013	− 0.018***	− 0.025**	0.015
	(0.007)	(0.007)	(0.011)	(0.007)	(0.010)	(0.011)
Household income	0.153***	0.030**	− 0.029	0.152***	0.044***	− 0.030
	(0.012)	(0.013)	(0.018)	(0.012)	(0.017)	(0.023)
Urban	0.420***	0.134***	− 0.128**	0.415***	0.227***	− 0.054
	(0.026)	(0.027)	(0.050)	(0.026)	(0.036)	(0.068)
County penetration	1.143***			1.257***		
	(0.076)			(0.079)		
Constant	− 4.076***	0.237	1.871***	− 4.155***	− 0.915***	1.628***
	(0.215)	(0.213)	(0.445)	(0.214)	(0.340)	(0.413)
Year	Yes	Yes	Yes	Yes	Yes	Yes
Province	Yes	Yes	Yes	Yes	Yes	Yes
$rho_{1}$		0.053***			0.499***	
		(0.029)			(0.140)	
$rho_{0}$			− 0.563***			− 0.363***
			(0.040)			0.192
Log pseudolikelihood	− 41,349.336	− 16,363.077				
Wald/LR test of indep. Eqn	chi2(2) = 120.82 Prob > chi2 = 0	chi2(2) = 10.81 Prob > chi2 = 0.005				
Observations	19,317			19,317		

∗ ∗  ∗, ∗  ∗ and ∗ denote significance at the 1% level, 5% level and 10% level, respectively

Standard errors are presented in parentheses

As shown in column (1), which is derived from Eq. (1), The coefficients in the selection equation elucidate the impact of demographic and socioeconomic factors on the likelihood of Internet use. Notably, age, gender, education, exercise, household income per capita, urban residence, and county penetration exhibit significant effects, signifying their roles in influencing the decision to engage with the Internet. The findings of this study suggest that, with increasing age and educational attainment, the likelihood of Internet usage rises. Boys exhibit a higher propensity for Internet use compared to girls. Additionally, per capita household income and urban residence indicate that higher incomes and more developed places of residence have a positive effect on Internet use. Column (4) is also derived from Eq. (1), and its results are similar to those in column (1), indicating consistency in the estimation of the factors influencing Internet use.

Moving to the outcome equations for BMI-for-age z-score and overweight, columns (2) and (5) report results for Internet users, derived from Eq. (2), while columns (3) and (6) present estimates for non-Internet users, based on Eq. (3). For both groups, age and gender demonstrate significant associations with BMI-for-age z-score and overweight. The consistency in the direction and significance of these variables across Internet users and non-users underscores their pervasive influence on health outcomes.

The treatment effects are calculated using Eqs. (4), (5), and (6). Table 3 presents the estimated effects of Internet use on adolescent nutritional health, showing that ATT is significant at the 1% level. The results indicate that if adolescents who currently use the Internet had not used it, their BMI-for-age z-score would decrease by 1.613, and their probability of being overweight would decrease by 0.102. The study further examines the impact of excessive Internet use and prolonged daily Internet use. Based on the average duration of Internet use, adolescents who spend more than two hours per day online during leisure time are classified as excessive users, a threshold commonly used in existing research. As shown in Table 3, excessive Internet use leads to a 1.531 increase in BMI-for-age z-score and a 0.146 increase in the probability of being overweight. Similarly, prolonged daily Internet use—defined as using the Internet for more than 2.147 h per day—results in a 1.541 increase in BMI-for-age z-score and a 0.147 increase in the probability of being overweight.

**Table 3:** The treatment effect of Internet use on adolescent nutritional outcomes *Sources*: The estimations are based on the CNHS 2006, 2009, 2011 and CFPS 2010, 2014, 2016, 2018, 2020, 2022

	Internet use	Excessive users (> 2 h per day)	Prolonged daily Internet use inferred from mean sample
	(1)	(2)	(3)
BMIZ	1.613***	1.531***	1.541***
	(0.005)	(0.009)	(0.008)
Overweight	0.102***	0.146***	0.147***
	(0.001)	(0.001)	(0.001)
Observations	19,317	17,483	17,483

∗ ∗  ∗, ∗  ∗ and ∗ denote significance at the 1% level, 5% level and 10% level, respectively

Standard errors are presented in parentheses

### Heterogeneity analysis

There remains a significant disparity in Internet infrastructure between urban and rural areas in China, despite ongoing public efforts to bridge this gap. Additionally, gender differences during adolescence can lead to varying growth patterns, influencing BMI-for-age z-scores. To assess the necessity of subgroup analysis, we conduct t-tests on the outcome and control variables using urban–rural and gender classifications (Table 12 and Table 13). The results indicate significant differences between groups, justifying the need for separate analyses. To further examine the impact of urban–rural disparities and gender differences on adolescent nutritional health, we employ the ESR model to analyze distinct subgroups within the sample.

As shown in Table 4, Internet use is associated with increased BMI-for-age z-score and a higher probability of being overweight across all subgroups. Rural adolescents experience a larger increase in BMI-for-age z-score at 1.710 compared to 0.492 for urban adolescents, while the probability of being overweight is slightly higher for urban adolescents at 0.072 than for rural adolescents at 0.044. For gender differences, both boys and girls show significant positive effects, with boys having a BMI-for-age z-score increase of 1.719 and an overweight probability of 0.115, while for girls, these values are 1.701 and 0.086, respectively.

**Table 4:** Heterogeneity impact on adolescent nutritional outcomes *Sources*: The estimations are based on the CNHS 2006, 2009, 2011 and CFPS 2010, 2014, 2016, 2018, 2020, 2022

	BMIZ	Overweight	Observations
	(1)	(2)	(3)
Urban	0.492***	0.072***	6377
	(0.009)	(0.002)	
Rural	1.710***	0.044***	12,940
	(0.007)	(0.001)	
Boys	1.719***	0.115***	10,143
	(0.010)	(0.001)	
Girls	1.701***	0.086***	9174
	(0.006)	(0.001)	

∗ ∗  ∗, ∗  ∗ and ∗ denote significance at the 1% level, 5% level and 10% level, respectively

Standard errors are presented in parentheses

Calculated using the ESR model

### Potential Mechanisms Analysis

Table 5 illustrates potential mechanisms for the impact of the Internet on adolescent BMI-for-age z-score. The ESR model is employed to assess the results of channel variables. The results indicate that Internet use significantly decreases carbohydrate and fat intake while significantly increasing protein intake in adolescents. Examining specific food and soft drink intake, adolescents who use the Internet consume fast food 4.478 times more frequently and drink 0.091 L more soft drinks compared to their non-Internet-using counterparts.

**Table 5:** The effects of Internet use on potential channels *Sources*: The estimations are based on the CNHS 2006, 2009, 2011

Variables	ATT	Observations
Carbohydrate	− 0.077***	1834
	(0.009)	
Fat	− 0.057***	1834
	(0.011)	
Protein	0.199***	1834
	(0.007)	
Fast food	4.478***	1834
	(0.037)	
Soft drink	0.091***	1834
	(0.017)	

∗ ∗  ∗, ∗  ∗ and ∗ denote significance at the 1% level, 5% level and 10% level, respectively

Standard errors are presented in parentheses

Calculated using the ESR model

### Robustness tests

We validate the robustness of the results by using two separate databases and applying the same methodology and variables. As shown in Table 6, the estimation results for both CHNS and CFPS data are positive and significant, which aligns with the overall findings. However, the BMI-for-age z-score estimation results for CHNS are smaller than the overall results and the CFPS results. This difference might be due to the CHNS sample data being from an earlier period (2006 to 2011), while the CFPS data is from a more recent period (2010 to 2022). With the rapid development of mobile Internet over time, especially the rise of mobile social entertainment software like short videos, the impact of Internet use on adolescents’ BMI-for-age z-score and overweight has become more significant.

**Table 6:** Main results robustness (ATT): separate estimations by data source *Sources*: The estimations are based on the CNHS 2006, 2009, 2011 and CFPS 2010, 2014, 2016, 2018, 2020, 2022

	BMIZ	Overweight	Observations
	(1)	(2)	(3)
CHNS	0.650***	0.100***	1834
	(0.025)	(0.004)	
CFPS	1.746***	0.102***	17,483
	(0.005)	(0.001)	

∗ ∗  ∗, ∗  ∗ and ∗ denote significance at the 1% level, 5% level and 10% level, respectively

Standard errors are presented in parentheses

Within the ESR framework, we apply the Mundlak correction to control for unobserved heterogeneity by incorporating the individual-specific means of time-varying covariates into both the selection and outcome equations [45]. This approach mitigates omitted variable bias by accounting for time-invariant individual characteristics that may jointly influence Internet use and health outcomes. Recent studies have successfully applied this method in similar contexts, supporting its relevance to our analysis [46, 47]. Our results remain consistent under this specification, further reinforcing the robustness of our findings (Table 7).

**Table 7:** ESR model with Mundlak correction *Sources*: The estimations are based on the CNHS 2006, 2009, 2011 and CFPS 2010, 2014, 2016, 2018, 2020, 2022

	Internet use	Internet overuse (> 2 h per day)	Prolonged daily Internet use inferred from mean sample
	(1)	(2)	(3)
BMIZ	1.594***	1.531***	1.536***
	(0.005)	(0.009)	(0.008)
Overweight	0.097***	0.146***	0.147***
	(0.001)	(0.001)	(0.001)
Observations	19,317	17,483	17,483

∗ ∗  ∗, ∗  ∗ and ∗ denote significance at the 1% level, 5% level and 10% level, respectively

Standard errors are presented in parentheses

For the third part of the robustness checks, we employ the Heteroskedasticity-Based IV approach proposed by Lewbel [48]. This method is particularly useful when conventional IVs are unavailable or weak, as it exploits heteroskedasticity in the data to generate valid instruments and has been increasingly applied in recent studies [49, 50]. Specifically, this approach addresses the endogeneity of Internet use by constructing an internal instrument based on the variance of residuals, ensuring that the identification of causal effects is not solely dependent on external IVs. The instruments are generated by interacting mean-centered covariates with the heteroskedasticity-driven residual variation from the first-stage regression, following Lewbel’s methodology.

Table 8 presents the results obtained from this alternative IV estimation. The Kleibergen-Paap rk LM and Wald F-statistics confirm the relevance of the instrument and reject the null hypothesis of weak instruments, while the Hansen J statistic suggests that the overidentification restrictions are not rejected, indicating that the instrument is valid. The key coefficients of interest correspond to the Internet variable, which remains positive and significant for both BMI-for-age z-score (column 1) and overweight status (column 2), aligning with the main results in Table 3. However, the coefficients are slightly smaller than in the main results, which may be due to the Lewbel IV approach incorporating additional variation through constructed instruments, leading to more conservative estimates.

**Table 8:** Alternative IV estimates: Lewbel's Heteroskedasticity-Based IV approach *Sources*: The estimations are based on the CNHS 2006, 2009, 2011 and CFPS 2010, 2014, 2016, 2018, 2020, 2022

	ZBMI	Overweight
	(1)	(2)
Internet	0.469***	0.067**
	(0.122)	(0.033)
Age	− 0.074***	− 0.025***
	(0.007)	(0.002)
Gender	0.209***	0.097***
	(0.020)	(0.005)
Education	− 0.015**	− 0.005***
	(0.007)	(0.002)
Exercise	− 0.008**	− 0.002***
	(0.003)	(0.001)
Household size	− 0.007	− 0.001
	(0.006)	(0.002)
Household income	0.013	− 0.002
	(0.011)	(0.003)
Urban	0.074***	0.018**
	(0.027)	(0.007)
Constant	0.616***	0.495***
	(0.181)	(0.051)
Year	Yes	Yes
Province	Yes	Yes
Observations	19, 317	19, 317
R-squared	0.054	0.079
Kleibergen-Paap rk LM statistic	287.765	287.765
Kleibergen-Paap rk Wald F statistic	18.142	18.142
Hansen J statistic	0.155	0.155

∗ ∗  ∗, ∗  ∗ and ∗ denote significance at the 1% level, 5% level and 10% level, respectively

Standard errors are presented in parentheses

## Discussion

The main goal of this paper is to examine the impact of Internet use on adolescent nutritional outcomes, exploring the pathways through which the Internet affects nutritional intake and dietary patterns. To the best of our knowledge, this is the first study to use BMI-for-age z-score as a criterion for adolescent nutritional outcomes, utilizing a large sample of long-term follow-up data. Our findings indicate that Internet use significantly increases the likelihood of higher BMI-for-age z-score and being overweight among adolescents. Specifically, more intensive leisure-time Internet use, whether above average or excessive, markedly raises the likelihood of increased BMI-for-age z-score and overweight, with the effect becoming more pronounced with greater use intensity. These results align with previous studies showing a connection between excessive Internet use and an increased likelihood of being overweight among adolescents [13, 51]. Our study estimates that Internet use makes adolescents 10.2% more likely to be overweight, with excessive use increasing this likelihood by 14.6%, figures that are more precise and smaller than those reported in earlier cross-sectional studies [12]. Although the overall impact of Internet use on adolescents appears negative, we do not oppose its use. Instead, we emphasize the need to understand the heterogeneity of different situations and mechanisms of action to better utilize the Internet for positive outcomes.

The urban–rural divide in adolescent health behaviors has been widely discussed in previous literature, with urban adolescents generally having better access to exercise facilities and health-related information [52]. However, our results suggest that these advantages do not necessarily translate into healthier weight outcomes, as both urban and rural adolescents experience similar risks of overweight due to Internet use. This aligns with research indicating that prolonged screen time and increased exposure to unhealthy food advertising may outweigh the potential benefits of health-related Internet use [53]. Similarly, our gender-based analysis reveals that boys and girls are equally affected by Internet use in terms of BMI and overweight likelihood. While previous studies suggest that boys may be more prone to excessive screen time, particularly for gaming [54], and that girls may be more influenced by body image concerns and dietary behaviors [55], our findings indicate that the overall impact on weight outcomes remains comparable across genders.

These results highlight the widespread risks associated with adolescent Internet use, regardless of location or gender, emphasizing the need for targeted interventions. This study analyzes the mechanisms through which Internet use affects adolescent nutritional outcomes from two perspectives: nutritional intake structure and dietary patterns. The findings indicate that Internet use significantly increases protein intake, as well as the frequency of fast food and soft drink consumption. Existing research highlights that excessive Internet use is often accompanied by unhealthy eating patterns and poor nutrition among adolescents [12, 32, 33, 56]. A plausible scenario is that during online gaming and video watching, adolescents develop unhealthy eating habits, tend to overconsume snacks, opt for high-calorie fast food, and consume large amounts of soft drinks [57, 58].

In contrast, studies on adults and older individuals suggest that Internet use can have positive health effects, including lower BMI, improved self-rated health, and better mental well-being. Prior research indicates that adults often utilize the Internet to enhance their dietary knowledge, seek online medical consultations, and access health-related resources, which contribute to better health outcomes [21, 24, 53]. Unlike adolescents, who primarily engage with the Internet for entertainment and social interaction, adults are more likely to use it as a tool for health management. This contrast underscores the importance of guiding adolescent Internet use toward more health-promoting behaviors, such as leveraging digital resources for dietary education and active lifestyle choices. Future research could explore ways to integrate health-oriented Internet engagement into adolescent digital habits.

Despite utilizing multi-period longitudinal data and conducting a study with a large sample size, this research has several limitations. First, although efforts were made to update the sample data as much as possible, it is not the most recent. Future research could address this by using the latest data to investigate whether there have been any changes in recent years. Second, the study did not differentiate between the purposes of Internet use during adolescents’ leisure time, failing to capture the nuanced effects of different Internet usage purposes on other adolescent behaviors. Future research could refine the classification of adolescents’ leisure time Internet use, such as online gaming and video watching, browsing, and socializing. Lastly, due to a lack of measurement of adolescents’ dietary knowledge in the data, this mechanism has not been validated. Future studies could explore the impact of Internet use on adolescents’ dietary knowledge.

## Conclusions and policy implications

The rising prevalence of overweight and obesity among adolescents has become a pressing issue, significantly impacting future workforce development. This study utilizes data from the CHNS and the CFPS to explore the effects of Internet use on adolescent nutrition-related health. Employing the ESR model to address biases caused by observable and unobservable factors, the research findings indicate a significant impact of Internet use on adolescent nutritional health. Specifically, Internet use among adolescents increases their BMI-for-age z-score by 1.613 and the likelihood of being overweight by 10.2%.

In light of these findings, specific measures are needed to address the issue. First, educational campaigns and public awareness initiatives should be launched to guide adolescents towards healthier Internet use, emphasizing the importance of balancing online activities with physical exercise and proper nutrition. Additionally, effective intervention strategies should be designed to mitigate the negative impacts of Internet use on adolescent health. These might include programs to reduce the consumption of soft drinks and high-calorie fast foods during online activities.

Our research identifies three primary channels through which Internet use affects adolescents’ nutritional health: increasing protein intake, elevating the frequency of fast-food consumption, and intensifying soft drink consumption. Importantly, Internet use is shown to reshape the daily eating habits of adolescents, fostering a preference for calorie-rich and sugary foods. In addition to intervening in adolescents’ Internet use, other avenues can also help them develop healthy eating habits. Drawing on international experiences, policies from other countries addressing similar issues should be considered. Successful intervention measures promoting balanced diets and active lifestyles among adolescents can serve as models for effective policy design, such as more effective food labeling [59]. By incorporating these strategies, more forward-thinking and comprehensive policy recommendations can be developed.

This research holds profound implications for adolescent health in an era where these issues are increasingly serious. At the governmental and societal levels, there is a need to advocate for healthy and responsible Internet use to prevent adolescent Internet addiction. Initiatives should be undertaken to foster healthy eating habits. At the school and parental levels, efforts should be made to regulate young people’s Internet usage, discouraging prolonged screen time after school. Adolescents themselves must be mindful of avoiding extended computer use and prioritize healthier dietary choices to cultivate positive eating habits. By implementing these targeted measures, leveraging international best practices, and promoting a holistic approach to health education, we can better address the adverse effects of Internet use on adolescent health and support the development of a healthier, more productive future generation.

## Data Availability

The data used in this study can be obtained free on the Internet, and the data sources have been given in the content. The data that support the findings of this study are publicly available. The China Family Panel Studies (CFPS) data can be accessed at https://www.isss.pku.edu.cn/cfps/en/, and the China Health and Nutrition Survey (CHNS) data can be accessed at https://www.cpc.unc.edu/projects/china.

## Appendix

See Tables

**Table 9:** Sample composition

Province	2006	2009	2010	2011	2014	2016	2018	2020	2022	Total	Database
Beijing	0	0	16	74	13	21	10	2	6	142	CHNS/CFPS
Tianjin	0	0	22	0	19	21	18	15	24	119	CFPS
Hebei	0	0	170	0	135	157	175	171	227	1,035	CFPS
Shanxi	0	0	232	0	119	111	106	110	120	798	CFPS
Liaoning	63	49	273	36	214	191	171	132	117	1,246	CHNS/CFPS
Jilin	0	0	58	0	46	43	34	26	29	236	CFPS
Heilongjiang	81	56	120	35	75	65	63	39	43	577	CHNS/CFPS
Shanghai	0	0	163	87	82	86	70	52	44	584	CHNS/CFPS
Jiangsu	58	59	74	59	41	38	27	34	37	427	CHNS/CFPS
Zhejiang	0	0	57	0	28	34	24	18	24	185	CFPS
Anhui	0	0	57	0	26	30	39	25	29	206	CFPS
Fujian	0	0	39	0	23	22	37	32	47	200	CFPS
Jiangxi	0	0	101	0	70	91	99	93	81	535	CFPS
Shandong	36	47	141	28	95	93	97	78	99	714	CHNS/CFPS
Henan	43	49	494	58	438	434	430	380	425	2,751	CHNS/CFPS
Hubei	50	45	54	34	35	39	32	29	23	341	CHNS/CFPS
Hunan	67	45	119	42	75	81	77	58	79	643	CHNS/CFPS
Guangdong	0	0	561	0	311	260	257	229	212	1,830	CFPS
Guangxi	72	107	93	116	77	83	74	66	84	772	CHNS/CFPS
Chongqing	0	0	39	79	18	40	27	21	28	252	CHNS/CFPS
Sichuan	0	0	208	0	116	149	184	137	142	936	CFPS
Guizhou	91	93	195	75	114	149	150	132	108	1,107	CHNS/CFPS
Yunnan	0	0	148	0	131	98	117	71	67	632	CFPS
Shaanxi	0	0	90	0	59	65	46	45	54	359	CFPS
Gansu	0	0	604	0	491	439	435	361	360	2,690	CFPS
Total	561	550	4,128	723	2,851	2,840	2,799	2,356	2,509	19,317	
Database	CHNS	CHNS	CFPS	CHNS	CFPS	CFPS	CFPS	CFPS	CFPS		

9,

**Table 10:** First-stage regression results for Internet use

	Internet use
County penetration	0.368***
	(0.019)
Age	0.031***
	(0.002)
Gender	0.042***
	(0.006)
Education	0.032***
	(0.002)
Exercise	0.004***
	(0.001)
Household size	− 0.004*
	(0.002)
Household income	0.041***
	(0.003)
Urban	0.115***
	(0.007)
Year	Yes
Province	Yes
Constant	− 0.688***
	(0.047)
Observations	19,317
Kleibergen-Paap rk Wald F statistic	369.451
Kleibergen-Paap rk LM statistic	365.816

Robust standard errors in parentheses

^***^ *p* < 0.01, ** *p* < 0.05, * *p* < 0.1

^10^,

**Table 11:** Reduced-regression of BMIZ and county penetration

	BMIZ	BMIZ	BMIZ	BMIZ
	(1)	(2)	(3)	(4)
County penetration	0.278***	0.073	0.277***	0.044
	(0.036)	(0.056)	(0.036)	(0.059)
Year	N	Y	N	Y
Province	N	N	Y	Y
Constant	− 0.479***	− 0.320***	− 0.018	0.021
	(0.018)	(0.058)	(0.113)	(0.130)
Observations	19,317	19,317	19,317	19,317
R-squared	0.003	0.011	0.037	0.043

Standard errors in parentheses

^***^ *p* < 0.01, ** *p* < 0.05, * *p* < 0.1

^11^,

**Table 12:** Descriptive statistics by urban and rural residence

Variable	All	Urban	Rural	Difference
	(1)	(2)	(3)	(4)
Internet	0.610	0.759	0.537	0.222***
	(0.488)	(0.428)	(0.499)	(0.007)
BMIZ	− 0.357	− 0.211	− 0.429	0.218***
	(1.339)	(1.341)	(1.333)	(0.020)
Overweight	0.149	0.180	0.133	0.047***
	(0.356)	(0.384)	(0.340)	(0.005)
Age	14.137	13.978	14.216	− 0.239***
	(2.826)	(2.846)	(2.813)	(0.043)
Gender	0.525	0.528	0.524	0.005
	(0.499)	(0.499)	(0.499)	(0.008)
Education	7.491	7.756	7.360	0.396***
	(2.845)	(2.902)	(2.807)	(0.043)
Exercise	3.280	3.535	3.154	0.381***
	(3.241)	(3.199)	(3.255)	(0.050)
Household size	4.798	4.273	5.057	− 0.784***
	(1.785)	(1.656)	(1.790)	(0.027)
Income	9.072	9.549	8.836	0.713***
	(1.135)	(1.108)	(1.072)	(0.017)
County penetration	0.439	0.499	0.409	0.090***
	(0.270)	(0.248)	(0.275)	(0.004)
Observations	19,317	6,377	12,940	19,317

Standard deviations in parentheses

^***^ *p* < 0.01, ** *p* < 0.05, * *p* < 0.1

^12^ and

**Table 13:** Descriptive statistics by gender

Variable	All	Boys	Girls	Difference
	(1)	(2)	(3)	(4)
Internet	0.610	0.631	0.587	0.044***
	(0.488)	(0.483)	(0.492)	(0.007)
BMIZ	− 0.357	− 0.247	− 0.480	0.233***
	(1.339)	(1.456)	(1.186)	(0.019)
Overweight	0.149	0.196	0.096	0.101***
	(0.356)	(0.397)	(0.294)	(0.005)
Age	14.137	14.099	14.180	− 0.081**
	(2.826)	(2.825)	(2.827)	(0.041)
Education	7.491	7.420	7.570	− 0.150***
	(2.845)	(2.811)	(2.879)	(0.041)
Exercise	3.280	3.506	3.029	0.477***
	(3.241)	(3.296)	(3.161)	(0.047)
Household size	4.798	4.650	4.961	− 0.311***
	(1.785)	(1.725)	(1.836)	(0.026)
Income	9.072	9.112	9.027	0.084***
	(1.135)	(1.137)	(1.131)	(0.016)
Urban	0.330	0.332	0.328	0.004
	(0.470)	(0.471)	(0.470)	(0.007)
County penetration	0.439	0.440	0.436	0.004
	(0.270)	(0.269)	(0.271)	(0.004)
Observations	19,317	10,143	9,174	19,317

Standard deviations in parentheses

^***^ *p* < 0.01, ** *p* < 0.05, * *p* < 0.1

^13^
